# Can Establishment Success Be Determined through Demographic Parameters? A Case Study on Five Introduced Bird Species

**DOI:** 10.1371/journal.pone.0110019

**Published:** 2014-10-15

**Authors:** Ana Sanz-Aguilar, José D. Anadón, Pim Edelaar, Martina Carrete, José Luis Tella

**Affiliations:** 1 Department of Conservation Biology, Estación Biológica de Doñana, Consejo Superior de Investigaciones Científicas (CSIC), Sevilla, Spain; 2 Instituto Mediterráneo de Estudios Avanzados, Consejo Superior de Investigaciones Científicas - Universidad de las Islas Baleares (CSIC-UIB), Esporles, Islas Baleares, Spain; 3 Department of Biology, Queens College, City University of New York, New York, New York, United States of America; 4 University Pablo de Olavide, Sevilla, Spain; University of Lleida, Spain

## Abstract

The dominant criterion to determine when an introduced species is established relies on the maintenance of a self-sustaining population in the area of introduction, i.e. on the viability of the population from a demographic perspective. There is however a paucity of demographic studies on introduced species, and establishment success is thus generally determined by expert opinion without undertaking population viability analyses (PVAs). By means of an intensive five year capture-recapture monitoring program (involving >12,000 marked individuals) we studied the demography of five introduced passerine bird species in southern Spain which are established and have undergone a fast expansion over the last decades. We obtained useful estimates of demographic parameters (survival and reproduction) for one colonial species (*Ploceus melanocephalus*), confirming the long-term viability of its local population through PVAs. However, extremely low recapture rates prevented the estimation of survival parameters and population growth rates for widely distributed species with low local densities (*Estrilda troglodytes* and *Amandava amandava*) but also for highly abundant yet non-colonial species (*Estrilda astrild* and *Euplectes afer*). Therefore, determining the establishment success of introduced passerine species by demographic criteria alone may often be troublesome even when devoting much effort to field-work. Alternative quantitative methodologies such as the analysis of spatio-temporal species distributions complemented with expert opinion deserve thus their role in the assessment of establishment success of introduced species when estimates of demographic parameters are difficult to obtain, as is generally the case for non-colonial, highly mobile passerines.

## Introduction

Birds are amongst the best studied taxa in the world. Given the historically large amount of data collected by scientists, environmental managers and amateur ornithologists, avian invasions have received much attention in the scientific literature especially through comparative studies (see review in [1]). For example, meta-analyses performed to evaluate factors influencing establishment success of introduced species have revealed that generalist species with broad niches, plastic behavior and slow life histories were more successful at establishing exotic populations (e.g., [1]–[3]). The validity of these comparative studies relies largely on the accurate determination of establishment success of the species. This seems to be an easy goal for those species deliberately introduced in past centuries, from which the fate of old introductions can be easily assessed nowadays [4], [5]. However, more recent introductions often resulted from the accidental escape of pet cage birds [6], which is increasing worldwide and leads to a contemporary processes of invasion [7]. This ongoing introduction of exotic birds [7] severely limits the temporal window offered by old introductions to assess whether a species is established or not.

Introduced species, including birds, are typically considered as established in their novel habitats when they maintain self-sustaining populations [8], [9]. This definition implies that the population is viable from a demographic point of view, i.e. that individuals survive and reproduce at sufficient rates to achieve a stable or growing population without the need of additional inputs [10], [11], and has been adopted by some countries for the assessment of bird establishments (e.g., United Kingdom [12]). Other criteria used to define the establishment success of introduced bird species also rely on demographic parameters, such as their reproduction in the novel habitat by more than 5 females [13] or their reproduction during a time period covering at least three generations [14]. Finally, some authors defined an exotic species to be successfully established if its introduction resulted in the establishment of a persistent or probably persistent population [15], [16], or consider their persistence during large time periods defined by subjective expert criteria (e.g. 25 years, [14]; 20 years, [3]; 15 years, [17]). The study of the demography of exotic species is thus essential to determine the fate of their recent introductions, as well as to assess evolutionary changes of life histories and the dynamics and future viability of their populations [10], [11], [18]–[20]. Life history studies and demographic models would also be very valuable for examining the population biology of introduced species and for identifying life history stages where management will be most effective [19], [21].

Nevertheless, although most definitions of establishment success imply demographic processes, very few studies have focused on the demography of introduced birds in their novel habitats [21]–[24] to obtain the survival and breeding parameters needed for assessing their population viability. In fact, data on demographic parameters is surprisingly scarce compared to the scientific attention devoted to avian invasions [1]. The most comprehensive database on vital rates of successfully introduced species to date [3] is actually composed of estimates from studies in captivity or in the native range, but not from the invaded areas. There is little doubt that differences in environmental conditions (e.g., climate, resources, competitors, predators, etc.) between native and non-native ranges could easily generate differences in demographic parameters [1]. In addition, introduced individuals might be under selection during the invasion stages of capture/uptake, transport, captive breeding and release/escape/introduction [25], [26], resulting in populations with potentially different demographic parameters relative to their native counterparts. Finally, data on vital rates are very scarce even for some very common groups of birds; for example, survival estimates were available for only 5 out of the 61 introduced species of the superfamily Passeroidea listed in Sol et al. [3].

Here we tested the validity of demographic criteria for assessing the establishment success of introduced exotic species, requiring the study of key demographic parameters, capture-mark-recapture modelling and population viability analysis [11], [20], [27]. We used as study models one Asian and four Sub-Saharan African passerines and introduced on the Iberian Peninsula (Spain and Portugal, SW Europe). These species are suitable for our approach since they are considered established; they are included in the Spanish Catalogue of Invasive Alien Species, and their possession, release and commercial trade thus forbidden (Real Decreto 630/2013). The inclusion of these species in the catalogue basically relied on expert assessment given that demographic analyses were not available for almost any of the species assessed (J.L. Tella, *personal observation*). Our specific objectives were: i) to provide estimates of population size, survival probabilities, and average lifespan of the introduced species in their novel habitats when possible, ii) to evaluate the efficacy of the demographic approach to determine the establishment success of exotic populations (i.e., the existence of viable populations) through population viability analyses.

## Methods

### Ethics Statement

Capture and banding of birds was conducted by expert bird-banders with permission from the government of Andalucia (complying with Real Decreto 1201/2005) and from the Ethics Committee of Estación Biológica de Doñana - Consejo Superior de Investigaciones Científicas (CEBA-EBD-11-27).

### Study species

We studied the Common Waxbill *Estrilda astrild*, the Black-rumped Waxbill *E. troglodytes*, the Yellow-crowned Bishop *Euplectes afer*, the Black-headed Weaver *Ploceus melanocephalus,* and the Red Avadavat *Amandava amandava*. They are small ubiquitous passerines native to tropical and southern Africa (waxbills, bishops and weavers) and tropical Asia (avadavats) with very large natural geographical ranges [28]–[30]. These species typically inhabit open country with tall grass, reed stands near water, cultivated areas, forest edges and the vicinity of human habitations [29], [30]. While the Black-headed Weaver is a colonial species during the breeding period, the other species do not breed in colonies but do concentrate in flocks during the non-breeding season [29]. In the Iberian Peninsula, these introduced passerine species have extended breeding periods (from April to November, *Authors’ unpublished data*). The Common Waxbill in the Iberian Peninsula can have several broods with 5 to 7 chicks per brood [31]. To date no reproductive information is available for the other species in their non-native areas, but clutch size in weavers and avadavats ranges from 2 to 6, usually 2–3, in their native areas [29], [30].

The five studied species were widely available in pet markets until 2005, and frequent accidental escapes of wild-caught individuals, rather than deliberate releases, seem to explain multiple past introduction events of these species in Spain ([6], *Authors’ unpublished data*). Since 2005 a European ban on trade in wild-caught birds stopped their imports [6], and nowadays they are just anecdotally kept and bred in captivity (*Authors’ unpublished data*).

### Spatial distribution

As part of a parallel study on the spatial and temporal distribution of bird species introduced in Spain and Portugal (*Authors’ unpublished data*), we compiled a large data set of exotic birds observed in the wild. For this goal we surveyed international, national and regional scientific journals, ornithological books and atlases, periodic regional publications (published and online), and a variety of ornithological internet forums where ornithologists usually communicate their observations and/or publish photographs of exotic birds. This information was complemented with personal communications of unpublished observations by expert ornithologists. All this information was checked for possible inaccuracies in the identification of species and double recording by different observers. This resulted in >13,000 records (involving ca 75,000 individuals) of >350 exotic bird species observed since 1912 (*Authors' unpublished data*). This included ca. 3,450 records (ca. 41,000 individuals) of the five species studied here. Given the demographic focus of this paper, we will just show here the spatial expansion in recent decades and the recent distributions of these species to illustrate the fact that they are established at a large spatial scale (Iberian Peninsula). Detailed analyses of spatio-temporal patterns of all the introduced species will be shown elsewhere (Authorś *unpublished data*).

### Field procedures

**Figure 1:**
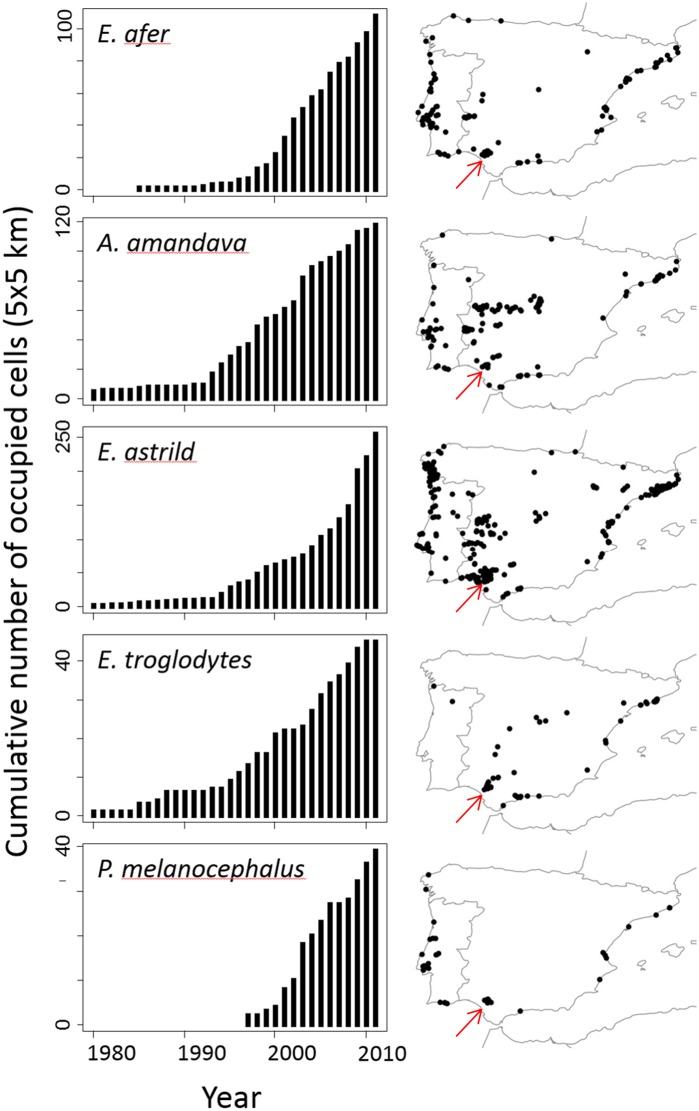
Temporal and spatial distribution of the study species on the Iberian Peninsula. Cumulative spatial distribution (left) and recent (year 2011) spatial distribution (right). In both cases, the spatial resolution is 5×5 km. The arrow indicates the study area where their demography was studied.

Our study areas lies in the lower Guadalquivir valley and in the surroundings of Doñana National Park (southern Spain, 36°56′51″N 6°21′31″O), a large marshland area (ca. 45,000 ha) where the five studied species are coexisting for long time (Fig. 1). From April 2008 to October 2012 three well-trained ornithologists devoted on average three days per week to locate and capture exotic birds in this study area. Each person independently searched for and caught exotic passerines with mist nets around wetlands and unharvested rice and maize crops throughout the year. The Black-headed Weaver, however, was more easily captured during the breeding period (April to September) in the surroundings of their colonies (but with a reduced capture effort in 2010 due to a personal accident). A total of 749 mist-net capture sessions were carried out on 623 days, and exotic birds were captured and marked with individually-numbered aluminum rings on 511 days. For each captured individual we recorded species, age and sex (when distinguishable by plumage or biometry), presence of brood patch (indicating active reproduction), and ring number when recaptured. Capture-recapture data are available under request.

### Recapture, survival and population size estimation

For each species we calculated total rates of recaptures of marked individuals and rates of recaptures with at least 6 months between first mark and subsequent recapture as an index of the chances of recapture at the medium term and thus the plausibility of obtaining robust annual survival estimates using capture-recapture analyses. Recapture probabilities, survival probabilities and population size were estimated by means of capture-recapture models [27]. Capture-recapture analysis began with a goodness-of-fit test of a model assuming complete time variation of recapture and survival parameters, the Cormack-Jolly-Seber model (CJS), to verify the assumptions of homogeneity in survival and recapture probabilities among individuals regardless of their past and present history [32]. This goodness-of-fit test is based on specific contingency tables for each recapture occasion and was calculated using the program U-CARE 2.3.2 [32]. For adult birds, Jolly-Seber capture-recapture models were built and fit to the data using the POPAN module [33] in the program MARK 6.0 [27]. POPAN models estimate time-specific catchability (*p*), survival rates (Ф), probabilities of entry into the population per sampling period (*pent*) that accounts for local recruitment and immigration, and population size (N) for open populations [33]. We tested for temporal variation in the parameters considered by performing models with time dependent (t) versus constant (.) formulations for Ф, *p* and *pent*. Model selection was based on the Akaike’s Information Criterion adjusted for small sample size and overdispersion (QAICc; [34]). Additionally for each model *j*, we calculated the Akaike weight, w*_j_*, as an index of its relative plausibility [34]. As in the CJS model, not all parameters are identifiable and only functions of parameters can be estimated in the fully time-dependent model (e.g. final survival and catchability or initial entrance and catchability). Using our obtained estimates of survival, average lifespan was calculated as −1/ln (adult survival) [35]. As the POPAN module does not permit the inclusion of age effects in the parameters of interest [33], we performed an additional analysis on birds captured as juveniles to estimate first year survival using CJS models in the program MARK 6.0 [27]. Additionally, using the estimated values of adult population size (N_t_) we calculated the annual population growth rate (λ_t_) as λ_t_ = N_t+1_/N_t_. We calculated the stochastic population growth (λ_s_) during the study period and its confidence interval by means of a linear regression procedure (see details in [10]). This method allows λ_s_ estimation by regressing the log population growth rate over a time interval against the amount of time elapsed [10].

In the winter of 2009–2010 bishops greatly concentrated during a short time period (two months) in a very reduced spatial area (an unharvested rice field), a special situation which allowed us to calculate their population size by means of closed capture-recapture models using the program MARK [36], [37]. One month elapsed between first and last captures, including six trapping occasions. As immediate recaptures could be influenced by individual behavioral trap responses [36], we considered four candidate models differing in capture probabilities: Model M*_0_*, a constant model; M*_t_*, a temporal model; M*_b_* a behavioral trap response model; and M*_tb_* a model accounting for temporal and behavioral effects [36]. Model selection was based on the AIC and population size (N) was estimated by model averaging [37].

### Breeding success estimation

Data on reproductive output of Black-headed Weavers in terms of number of fledglings per brood was collected by nest monitoring during the 2011 breeding season. We accessed nests from a boat, and used small numbered metal labels to individually mark the branch that supported each nest. This branch was cut, since the nest shape otherwise did not allow the reliable assessment of number of eggs and chicks, and afterwards the branch was attached again with the use of plastic tie-wraps. Only a very low proportion of nests failed (3.5%, 1 out of 28 nests) which may well represent the normal failure rate. Moreover, four such manipulated nests had new eggs after chicks had fledged, so we believe that this method did not cause unacceptable levels of disturbance and bias in our data. The colony was visited four times, from 27 July to 21 Sept. 2011. Broods of large feathered chicks close to fledging whose nests were empty and undamaged at return visits were assumed to have fledged successfully, yielding number of fledglings per brood. We never found large dead chicks in the nest, so we think this is a reasonable assumption. Additionally, for Black-headed Weavers we calculated the ratio between juvenile individuals and adult females captured with mist-nests around the breeding colony towards the end of the breeding season (i.e., July to October, when many chicks have fledged) for each year. Juveniles were identifiable by their fresh plumage and eye color. This ratio can be considered a measure of breeding success when probabilities of capturing - adults and juveniles are similar [38]. Unfortunately, in our case the breeding season last from April to October and adult females have higher chances of being captured during the breeding season than juveniles, since the latter abandon the colony soon after fledging while females stay for successive breeding attempts (Authors’ personal observations). However, the annual variation in this ratio was used as a proxy of between-year variability in breeding success (see below).

### Population matrix modelling

The analysis presented here was only carried out for the Black-headed Weaver due to difficulties in estimating demographic parameters in the other species considered (see results). Age-structured stochastic matrix population models were built to forecast stochastic population growth rate λ_s_ and calculate extinction probabilities under different scenarios of fecundity using the program ULM [10], [20], [39]. Using the yearly estimates of juvenile and adult annual survival rates and their standard errors, we applied White’s method to obtain estimates of the temporal variance and simulate the environmental stochasticity using a beta distribution [10]. Demographic stochasticity was also included in the population projections; the Poisson distribution was used for fecundity and the binomial distribution for survival. Density-dependence (either positive or negative) was not included in models because no evidence of Allee effects or carrying capacity limitation was available for the study population and also because we were not interested in the estimation of the final number of individuals at the end of the projections. Ten thousand Monte Carlo runs of stochastic population models were simulated over a 50-year period and mean stochastic population rates over trajectories, λ_s_, for each combination of demographic parameters (see below) were calculated [10], [20]. In a stable population, the population growth rate is equal to 1, higher values characterise an increasing population and lower values a decreasing population [10], [20]. The initial breeding population value used in the simulations was the mean breeding population estimated during the study period by capture-recapture modelling. Only the female population was modelled and it was assumed that sex ratio at birth was 0.5 (Authors’ personal observation). We set age at first breeding for females at one year (*Authors’ unpublished data*) and assumed that survival was not sex-specific. Regarding the number of broods per year, the presence of active incubation patches in female Black-headed Weaver captured and recaptured during the whole breeding season confirmed that they are able to produce 3 broods due to the extensive breeding season in the study area (April to October, *Authors’ unpublished data*). Our estimates of breeding success were based on relatively little data (see above), so we simulated the effect of parameter uncertainty by considering different possible values (ranging 2.12 to 4.94 female fledglings by adult female, see below). In the lower limit we considered 0.5 sex ratio, two clutches per female and the mean breeding success as estimated in 2011 (when the ratio between juveniles and adult females was lowest and equal to 1; 0.5*2*2.125*1 = 2.125). In the upper limit we considered 0.5 sex ratio, 3 clutches per female and the highest estimated breeding success across years (when the ratio between juveniles and adult females was 1.55 times higher as in 2011; 0.5*3*2.125*1.55 = 4.94).

Additionally, age-structured deterministic matrix population models for the different combinations of demographic parameters were built to calculate the plausible ranges of generation time, T, defined as the time required for the population to increase by a factor equals to its net reproductive rate [20].

## Results

### Spatial distribution

The five exotic species differ in their time of first introduction: the Common Waxbill was first introduced in the 60′s in Portugal, the Red Avadavat and the Black-rumped Waxbill were first introduced in Portugal and Spain in the 70′s, the Yellow-crowned Bishop in the 80′s in Spain, and the Black-headed Weaver was first recorded in the mid 90′s in our study area. Despite these differences all five species show a remarkable similarity in the temporal evolution of their distribution over the last four decades (Fig. 1). Their range expansions and current widespread distributions justify their listing as established invasive species in the Spanish Catalogue of Invasive Alien Species (Real Decreto 630/2013) and their use in this study as study models to test whether establishment can be assessed through demographic criteria.

### Capture-recapture of individually marked individuals

**Figure 2:**
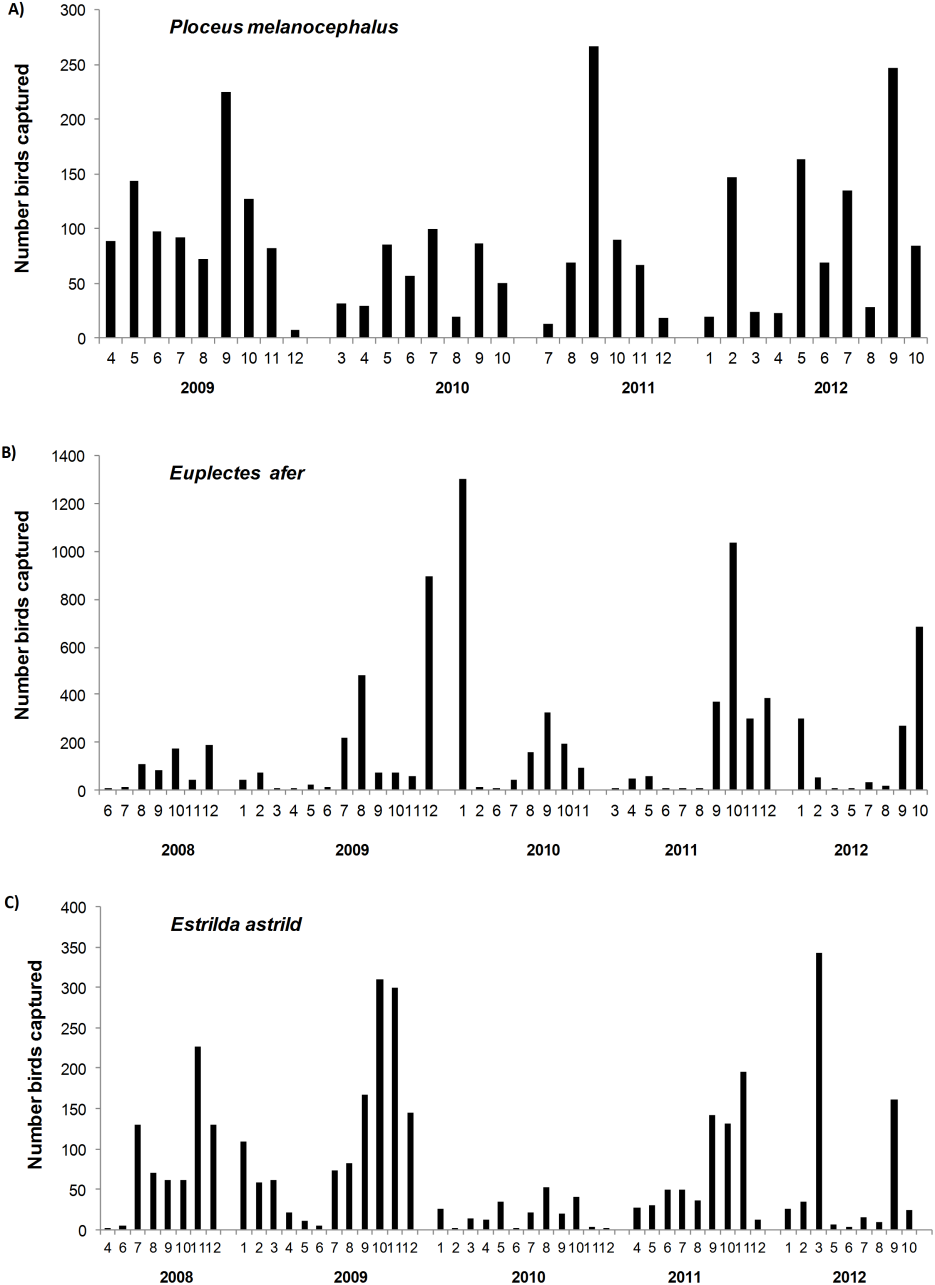
Monthly number of birds captured. (A) Black-headed Weaver *P. melanocephalus*, (B) Yellow-crowned Bishop *E. afer* and (C) Common Waxbill *E. astrild* captured during the study period in the study area (Doñana National Park area, Southern Spain).

**Figure 3:**
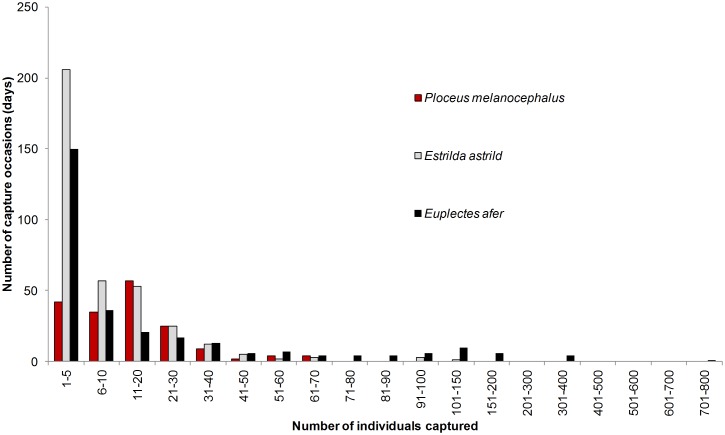
Histogram of more abundant exotic birds captured per mist-net occasion. Number of individuals of Black-headed Weaver *P. melanocephalus*, Yellow-crowned Bishop *E. afer* and Common Waxbill *E. astrild* captured per mist-net occasion.

The species under study showed a very high temporal variability in the number of captured individuals (Fig. 2). During most mist-net occasions, low numbers of birds were captured, although large numbers could be reached in a single occasion: up to 720 Yellow-crowned Bishops, 108 Common Waxbills and 70 Black-headed Weavers (Figure 3). The most abundant species, in terms of number of individuals captured, was the Yellow-crowned Bishop, followed by the Common Waxbill and the Black-headed Weaver (Table 1). The Black-rumped Waxbill and the Red Avadavat were captured in low numbers (Table 1). Additionally, three other exotic passerine species (*Estrilda melpoda*, *Lonchura punctulata* and *Quelea quelea*) were incidentally captured (Table 1). The Black-headed Weaver was the species with the highest recapture rates (Table 1), which contrasts with the very low percentages of recaptures after at least six months in the other species (Table 1). When considering wintering periods (November to February) in which bishops and Common waxbills aggregated (see Fig. 2), the percentage of recaptured individuals in subsequent winters was also very low (1.17% and 0.08%, respectively). Consequently, the only species with a sufficiently high proportion of recaptured individuals for robust open capture-recapture analysis to estimate demographic parameters (annual survival and adult population size) was the Black-headed Weaver. The Yellow-crowned Bishop and the Common Waxbill were mainly recaptured at the short term (Table 1) but there was a substantial temporal heterogeneity in the distribution of their captures and recaptures (Fig. 2, 3).

**Table 1:** Number (N) of captured and recaptured individuals per exotic species in the study area.

	N captured	N (%) recaptured	N (%) recaptured >6 months
*Ploceus melanocephalus*	1804	593 (32.87%)	268 (14.85%)
*Euplectes afer*	6844	882 (12.89%)	245 (3.58%)
*Estrilda astrild*	3139	209 (6.66%)	33 (1.05%)
*Estrilda troglodytes*	193	27 (13.98%)	3 (1.55%)
*Estrilda melpoda*	3	0	0
*Amandava amandava*	157	21 (13.38%)	1 (0.64%)
*Lonchura punctulata*	7	0	0
*Quelea quelea*	1	0	0

For recaptures, we give the totals and proportions for recaptured individuals independent of time between captures, and for those individuals recaptured at least 6 months after first capture.

### Demographic parameters and population viability of the Black-headed Weaver

Captures of adult (April to September) and juvenile (April to October) weavers within the same breeding season were pooled together to obtain a single capture-recapture occasion per year. We analysed 790 captures and 120 recaptures of adult individuals and 811 captures and 104 recaptures of birds marked as juveniles during the breeding seasons 2009–2012.

#### Adult survival

The overall test of goodness-of-fit of the CJS model was not statistically significant (χ^2^ = 42.60, d.f. = 40, P = 0.37). The variance inflation factor, ĉ, used in the analyses was 1.065. Models with recapture probabilities that varied in time were better supported than constant models (Σw*_j_* = 0.97; Table 2). The best model in terms of QAICc considered yearly variation in recapture probability and constant survival and entrance (pent) probabilities (w*_j_* = 0.38; Model 5, Table 2). Model 5 (Table 2) was fairly close in terms of QAICc to models 4 and 2 (Table 2) which considered additional temporal variation in entrance or survival probabilities, respectively. We only present the results of the best model (Model 5, Tables 2–3) because of its better fit and because in the other models several parameters such as the initial population sizes (Models 2 and 4, Table 2) or the last survival and recapture rates (Model 2, Table 2) are not separately identifiable [33]. Annual adult survival was estimated to be 0.50 on average (CI: 0.38–0.62, SE: 0.06, Model 5, Table 2); hence the estimate for average adult life span for the species was 1.44 years.

**Table 2:** Overview of POPAN capture-recapture models of adult Black-headed Weaver.

Model	*Ф*	*p*	*pent*	np	QAICc	w*_j_*
1	t	t	t	9	742.09	0.10
2	t	t	.	8	740.34	0.24
3	t	.	t	8	744.33	0.03
4	.	t	t	8	740.22	0.25
**5**	**.**	**t**	**.**	**7**	**739.42**	**0.38**
6	.	.	t	6	755.32	0.00
7	t	.	.	5	763.37	0.00
8	.	.	.	4	817.69	0.00

‘Φ’ = probability of survival; ‘*p*’ = probability of capture; ‘*pent*’ = probability of entrance in the population; np = number of parameters estimated; QAICc = Akaike information criterion corrected for small sample size and overdispersion; w*_j_* = Akaike’s model weight. Model notation: ‘t’ = time effect, ‘.’ = constant. The model with the highest support is in bold.

**Table 3:** Estimates of adult survival rate, annual recapture rates and annual population sizes for the Black-headed Weaver.

Parameter	Estimate	CI
Mean Ф	0.50	0.38–062
*p* 2009	0.56	0.14–0.91
*p* 2010	0.30	0.21–0.41
*p* 2011	0.18	0.12–0.27
*p* 2012	0.39	0.22–0.58
N 2009	514	224–1181
N 2010	655	476–902
N 2011	727	501–1053
N 2012	763	485–1199

‘Φ’ = probability of survival; ‘*p*’ = probability of capture (year 2009) and recapture (years 2010 to 2012); ‘N’ = population size; CI = 95% confidence intervals. Estimates were obtained from Model 5 (Table 2).

#### Juvenile survival

The overall test of goodness-of-fit of the CJS model considering two age classes in survival (i.e., different juvenile and adult survival, our general model) was not statistically significant (χ^2^ = 0.724, d.f. = 2, P = 0.70). The variance inflation factor, ĉ, used in the analyses was 1. Following the results of the previous analysis on adult birds, second year survival (i.e., adult) was considered constant in all models. Models with recapture probabilities that varied in time were better supported than constant models (Σw*_j_* = 1; Table 4). The best model in terms of AICc considered constant juvenile survival probabilities (w*_j_* = 0.62; Model 2, Table 4). Mean juvenile survival was 0.22 (CI: 0.15–0.30, SE: 0.04, Model 2, Table 4).

**Table 4:** Overview of capture-recapture models of juvenile Black-headed Weaver.

Model	*Ф*	*p*	np	AICc	w*_j_*
1	t	t	7	638.45	0.38
**2**	**.**	**t**	**5**	**637.46**	**0.62**
3	t	.	3	650.14	0
4	.	.	3	660.64	0

‘Φ’ = probability of juvenile survival; ‘*p*’ = probability of capture; np = number of parameters estimated; AICc = Akaike information criterion corrected for small sample size; w*_j_* = Akaike’s model weight. Model notation: ‘t’ = time effect, ‘.’ = constant. All models considered constant adult survival. The model with the highest support is in bold.

#### Breeding success

The mean number of fledglings per nest during the 2011 breeding season was 2.125 (n = 8 nests). Ratios of number of juveniles to number of females suggested that breeding success may vary between years, being the lowest in 2011 (Table 5). In 2009, 2010 and 2012 the ratio was 1.13, 1.55 and 1.40 times higher than in 2011, respectively, suggesting a higher breeding success in those years (Table 5).

**Table 5:** Ratios between numbers of juveniles and adult females of Black-headed Weavers captured towards the end of the breeding season (July–October), and estimated breeding success.

Year	2009	2010	2011	2012
Number of juveniles	217	133	134	226
Number of adult females	76	34	53	64
Ratio	2.86	3.91	2.53	3.53
Breeding success	2.402	3.284	*2.125*	2.965

Breeding success was measured in 2011 (see text) when the ratio was lowest (2.53), and breeding success in the remaining years was increased proportionally according to the higher juveniles/females ratios in each year.

#### Population viability

**Figure 4:**
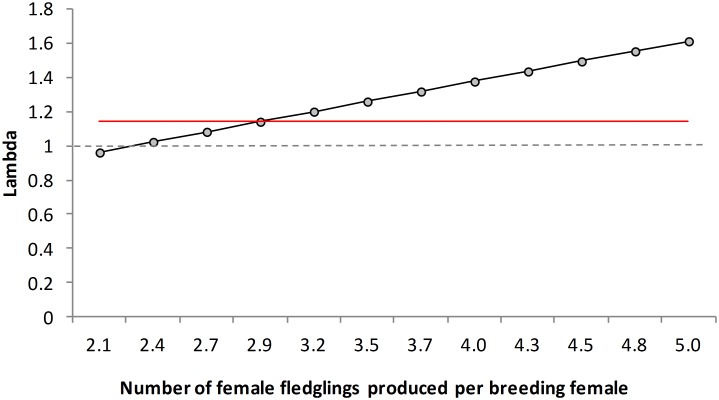
Stochastic population growth rates for the studied population of Black-headed Weavers. Stochastic population growth rate (λ_s_) under different potential numbers of female fledglings produced by breeding female across the entire breeding season. The value of lambda 1 (dashed grey line) indicates population stability. The red line indicates the stochastic population growth rate calculated using the count-based Population Viability Analysis.

Using these data as input, matrix population modelling indicated that with more than 4.5 fledglings (i.e. 2.25 females) per breeding female per breeding season populations always showed positive growth (Fig. 4), and extinction probability during the next 50 years was zero.

Generation time ‘T’ ranged from 1.147 to 2.034 years under the higher and the lower considered fertility estimates (i.e., combining breeding success and number of broods).

### Population size estimates, annual and count-based stochastic population growth rate

The low percentage of recaptures obtained from most species only allowed the estimation of population size through Jolly-Seber capture-recapture models for the Black-headed Weaver. The best-supported model (model 5, Table 2) estimated a population size during the breeding season that varied among years from 514 to 763 individuals. Annual population growth rates were 1.27 (2009 to 2010), 1.11 (2010 to 2011) and 1.05 (2011 to 2012). The estimated count-based stochastic population growth rate λ_s_ was 1.14 (1.02–1.28). However, the 95% CIs of population size largely overlapped across years (Table 3), giving limited confidence to the apparent changes in population size from year to year and in λ_s_.

During winter 2009–2010, thousands of Yellow-crowned Bishops concentrated on a single unharvested rice field within the study area. Surveys conducted outside this restricted area failed to detect foraging groups of the species during this period. This offered us an exceptional occasion to estimate population size through a closed capture-recapture design. Models M*_t_* and M*_bt_* were tied in terms of AIC (Table 6). The model averaged estimate of the winter population size of the species was 6036 individuals (CI: 4951–8477).

**Table 6:** Overview of closed capture-recapture models of Bishops.

Model	np	AICc	ΔAICc	w*_j_*
**M** ***_t_***	**7**	**−16221.84**	**0.00**	**0.57**
M*_bt_*	8	−16221.29	0.55	0.43
M*_b_*	3	−15324.62	897.22	0
M*_0_*	2	−15283.75	938.09	0

‘np = number of estimable parameters; AICc = Akaike information criterion corrected for small sample size; ΔAICc: the AICc difference between the focal model and the one with the lowest AICc value; w*_j_* = Akaike’s model weight. Model notation: ‘*t*’ = time effect, ‘*b*’ = behavioral trap effect, ‘*0*’ = no time of behavioural trap effect. The model with the highest support is in bold.

## Discussion

The most accepted criterion to classify an introduced species as successfully established is the maintenance of a self-sustaining population [8], [9], a clear definition that implies stable or growing populations (i.e., λ≥1) without any kind of human-assisted arrival of new individuals. However, few studies have focused on demographic parameters and population growth rates of introduced avian species [1]. Therefore, in the absence of accurate demographic information, a disparity of criteria and expert opinion have been commonly used to list species in the different catalogues of invasive species, thus resulting in substantial differences amongst them that could translate into inconsistencies among invasion studies and management policies. For example, introduced species are typically not identified as established or invasive until they have reached large numbers and have spread across a considerable area [40], and even in some cases only when they are causing proven impacts in the novel environment [41]. While clearly a more consistent application of criteria and the demographic approach would be preferred, our results illustrate the difficulty of applying demographic criteria for some species. Here, even with a very intensive and economically expensive long-term field survey, our demographic study allowed us to confirm as established only one of the five introduced species which by other criteria appear well-established in the study area and are widely distributed across the Iberian Peninsula (Fig. 1). Therefore, alternative quantitative criteria and interpretation of all available information by experts seems necessary to assess establishment when demographic information is very difficult to obtain due to the ecological characteristics of the species. Detailed spatio-temporal analysis of distributions of introduced species (Fig. 1) may reveal itself as an alternative, quantitative approach that can be used to assess the establishment and growth of populations ([42], *Authors’ unpublished data*). Spatio-temporal patterns of distribution must however be interpreted with caution, since they reflect a mixture of demographic and introduction processes. On the one hand, temporal lags between establishment and spatial spread (lasting sometimes decades) are common among introduced bird species [43]. Therefore, before spread, a population may have a positive population growth and its establishment could be confirmed through demographic criteria (e.g., population viability analysis) but not through spatial analyses. On the other hand, some species may apparently spread over large areas but not be truly established if the spread is due to multiple introduction events rather than to intrinsic population growth - in such cases the populations will vanish after stopping further releases. Therefore, the spatio-temporal approach needs to be complemented with knowledge from experts for a correct interpretation. Expert opinion is an important source of information for conservation and resource management decision making as experts can provide a synthetic perspective, drawing on their own observations and experience and all available published and unpublished data [44]. However, we would like to stress that expert opinion together with alternative quantitative approaches cannot fully replace the value of demographic studies -when feasible- to improve our understanding of the systems involved.

Demographic matrix models or even spatially explicit population models can be used to address both the successful establishment (i.e. viability) of exotic populations and to provide effective guidelines for their management and control [21], [24], [45]. Model performance is very sensitive to the accuracy of the life history parameter input and, thus, unfortunately, models can only be used for species with detailed data on demographic and population parameters [10], [11], [18], [20]. Spatial differences in environmental conditions and individual selection processes during the invasion stages may promote differences in demographic parameters between populations in native versus non-native ranges [1], [25], [26]. Demographic models on avian exotic species are usually based on estimates obtained from the specieś native range [3]. Consequently, the validity of the results of demographic models will depend on the demographic match between the native and novel range.

Demographic parameters and establishment success of exotic passerines. We could only reliably estimate demographic parameters, generation time, and population growth rate for the Black-headed Weaver. This is the first published survival estimate for this species, so comparison with the non-native range is problematic. Our estimate of local annual adult survival of 0.50 was lower than that found by Peach et al. (2001) for another weaver species in Malawi (0.70, *Ploceus xanthopterus* [46]) but similar to that found by McGregor et al. (2007) for Nigerian *Ploceus spp*. [47], and it is within the usual range of annual survival rates of passerines [3], [48]. A comparison with survival from the native range could test whether survival is reduced in favor of greater reproductive effort as could be expected for a growing population. Our estimate of juvenile survival (0.22) is within the normal range for sympatric native passerine species, with values ranging 15–30% [49], [50]. Contrarily to our results, studies on tropical passerines revealed that their juvenile survival is commonly higher [51]; for example in Sociable weavers (*Philetairus socius*) survival was similar between juveniles and adults [52]. The observed number of fledglings per brood (2.125) and number of broods (up to 3) are also within the usual range of African weavers [29], [53]. Unfortunately, unequal catchability of juveniles and adult females due to the extended breeding period and early juvenile dispersal prevented a reliable estimation of breeding success based only in juvenile/adult ratios of captured birds [38].

The estimates of local survival are probably lower than real values because they do not account for permanent emigration [11], [54]. However, most of the simulated plausible values of annual breeding success resulted in positive population growth rates (Fig. 4). Consequently, based on our demographic analyses we can confirm that this species has successfully established in the study area and is undergoing a process of population growth and expansion. This conclusion is supported by the fact that the observed annual population growth rate (based on count-based PVA) was higher than one (1.14, Fig. 4), and matched with a mean value of 5.8 fledglings produced per breeding female across the entire breeding season, which appears a quite realistic figure. On the other hand, the population seems to be self-sustaining without the existence of new input as the import of wild-caught weavers ceased in 2005 with the European wild-trade ban [6] and nowadays this species is sporadically kept in captivity (*own observation*), so the possibility of further accidental escapes which could reinforce the wild population is negligible. Conversely, some illegal trapping of weavers in the study area (*own observation*) might be currently slowing down population growth.

Additionally, the presence of the species at the study area was observed for more than 6.1 years (i.e., 3 times the species generation time, Fig. 1) confirming its establishment following the criteria of reproduction during a time period covering at least three generations [14].

Contrasting with the above case, and despite a great capture effort during the whole study period, the low recapture rates of waxbills, bishops and avadavats (Table 1) prevented the robust estimation of their local survival and annual population size. Consequently, it was not possible to perform population modelling to address and confirm the viability of their populations. We could only apply closed capture-recapture models to estimate the Yellow-crowned Bishop population size in a special situation in which the entire population seemed to aggregate at a single location. Our estimates indicated that a large population of several thousand individuals of this species occurs in the study area. The low rates of recaptures among waxbills, bishops and avadavats may be explained by two non-exclusive hypotheses: i) the existence of very large local populations which is supported by the high numbers of different individuals captured (Table 1), and ii) dispersal out of the study area which is supported by the existence of local movements (own observations) and nomadism described for bishop and waxbills populations [29]. These facts are not exclusive of exotic species, since low recapture rates also prevented the successful estimation of demographic parameters in several species of coexisting native passerines (*Authors’ unpublised data*).

### Practical considerations for the study and management of exotic species

Data collection for productivity or fecundity estimation is labor intensive, necessitating finding and monitoring nests and breeding attempts throughout the breeding season. Small birds, such as passerines, can be hard to detect and seldom stay in the same location between breeding occasions. Closed capture-recapture models to estimate population size assume that no reproduction, mortality, immigration or emigration occurs during the sampling periods [37]. This critical assumption cannot be achieved when birds distribute and move across large areas, and consequently individuals captured in the first occasion are not present in the sampling area in later capture occasions. In addition, most capture-recapture models assume that capture probability is constant across individuals [27], [54]. When individuals vary in their capture probabilities, the most catchable animals (for example, those breeding closer to mist nets) are likely to be caught first and more often. This leads to capture probability being overestimated and abundance being underestimated [27]. Although some capture-recapture models are able to deal with heterogeneity in capture probability, estimates of parameters of interest are not robust when recapture probability is small [55]–[57].

The assumption of homogeneous catchability is also crucial for Jolly-Seber open capture-recapture models: unmarked animals should have the same probability of capture as marked animals in the population [33]. This critical assumption prevents the use of open capture-recapture models to estimate survival and population size at the very short term [33] because birds tend to avoid nets once they have been captured [58]. This tendency is more pronounced among some species, especially tropical ones, as those considered in this study [58]. In addition, short term survival estimates may not reflect annual survival if survival varies over the year. However, by pooling capture data during the whole breeding season of the only colonial species considered, the Black-headed Weaver, its annual local adult survival and population size could be robustly estimated. The coloniality of the Black-headed Weaver facilitated their recapture and nest monitoring, and consequently the estimation of their demographic parameters, as was the case for another colonial introduced bird species [21]. However, the Black-headed Weaver is locally less abundant (around 700 adults) than other species such as the Yellow-crowned Bishop or Common Waxbill. Our estimate of Yellow-crowned Bishop population size (around 6,000 individuals) and the total number of both bishops and Common Waxbill captured suggest the existence of much larger local populations of these species than that of the Black-headed Weaver. Finally, the Red Avadavat and the Black-rumped Waxbill are present at low densities in the study area, thus precluding enough recaptures for demographic modelling. Hence, the ability to successfully use the demographic approach to assess establishment success depends critically on the biological characteristics and local density of each species affecting their recapture rates. In a similar vein, most capture-recapture based demographic studies of native population of birds come from colonial (e.g., seabirds) and highly territorial (e.g., raptors) species whose behaviors and spatial distributions facilitate high recapture rates (e.g., [49], [57]).

An alternative to capture-recapture approaches to study the demography of highly mobile bird species could be the use of radio-tracking techniques that may facilitate both the long-term monitoring of individual survival and the location of nests and monitoring of reproduction [59]. However, this is also a highly resource-consuming method and technically still unfeasible for the smallest of our study species.

Possibly for any or all of the above reasons, there is an absence of data on key demographic parameters for recently introduced birds, and most assessments of their establishment success rely on expert assessment (e.g., [12]). The difficulty with which we could obtain key demographic parameters supports the use of such alternative (though in many ways inferior) approaches to assess establishment success. It is worth noting that, although our study focused on small passerines, the feasibility of performing long-term capture-recapture studies and obtaining breeding parameters from other exotic vertebrate taxa (e.g., mammals, reptiles and fishes) can be even harder than for birds, making difficult to assess their establishment success on the basis of demographic criteria.

The difficulty of obtaining key demographic parameters also has repercussions for management and policy. A common feature of biological invasions is the lag time between initial colonization and the onset of rapid population growth and range expansion [60] and the identification of their potential impacts on native biota and ecosystems [60]. Limiting or reversing population growth of invasive species is usually hard to accomplish [40], [61], [62]. Investing more time and economic resources into obtaining better estimates of demographic parameters to perform population viability analyses and assess establishment and sensitivity of populations to control of certain life stages may delay their management, and during this time populations may grow and spread to such an extent that their control could become too difficult and expensive [61]. Consequently, the control of seemingly establishing populations should begin as soon as possible to avoid further potential ecological and economic costs [61].

### Conclusions

Our study shows that determining the establishment success of introduced passerine species by demographic criteria can be difficult and will depend on the biological characteristics, distribution and density of the species considered. These results support the validity and use of alternative procedures which are less methodologically constrained, such as the spatio-temporal analysis of species distributions complemented with more subjective expert criteria when demographic analyses are difficult to perform.

## Acknowledgments

We thank J. Ayala, A. Jurado, M. Vázquez, D. Serrano, J. Potti and J. Blas for their assistance in the field and M. Genovart, JD Lebreton, O Gimenez, D. Koons and A. Hernández-Matías for their advice in population modelling.
